# Shroom3 facilitates optic fissure closure via tissue alignment and reestablishment of apical-basal polarity during epithelial fusion

**DOI:** 10.1016/j.ydbio.2025.03.008

**Published:** 2025-03-18

**Authors:** Jessica A. Herstine, Jordyn Mensh, Electra Coffman, Stephanie M. George, Kenneth Herman, Jessica B. Martin, Ali Zatari, Heather L. Chandler, Zbynek Kozmik, Thomas A. Drysdale, Darren Bridgewater, Timothy F. Plageman

**Affiliations:** a College of Optometry, The Ohio State University, Columbus, OH, USA; b Institute of Molecular Genetics, Academy of Sciences of the Czech Republic, Prague, Czech Republic; c Department of Physiology and Pharmacology, Schulich School of Medicine & Dentistry, Western University, London, Ontario, Canada; d Department of Pathology and Molecular Medicine, Faculty of Health Sciences, McMaster University, Hamilton, Ontario, Canada

## Abstract

Optic cup morphogenesis is a complex process involving cellular behaviors such as epithelial folding, cell shape changes, proliferation, and tissue fusion. Disruptions to these processes can lead to an ocular coloboma, a congenital defect where the optic fissure fails to close. This study investigates the role of Shroom3, a protein implicated in epithelial morphogenesis, in mouse embryos during optic cup development. It was observed that Shroom3 is apically localized in the neural retina and retinal pigmented epithelium, and its deficiency leads to a both a conventional coloboma phenotype characterized by a gap in pigmented tissue as well as a unique type of coloboma where an ectopic ventral fold of neural tissue is present. Increased apical areas of both neural retina and retinal pigmented epithelial cells are present in the absence of Shroom3 leading to a greater apical surface area and disruption of optic fissure alignment. Neural retina specific gene ablation revealed that Shroom3 function in the RPE is likely sufficient to facilitate tissue alignment and permit fusion. However, the fusion process is ultimately disturbed due to a failure of the neural tissue to reestablish apical-basal polarity. Furthermore, it is demonstrated that Shroom3 deficiency also affects other epithelial fusion events in the embryo that rely on polarity reestablishment, such as lens vesicle separation, eyelid formation, and secondary palate closure. These findings highlight the importance of Shroom3 during optic cup morphogenesis, aid our understanding of optic fissure closure and coloboma formation, and implicates a role for Shroom3 in regulating apical-basal polarity.

## 1. Introduction

During early vertebrate retinal development, the morphogenesis of the optic vesicle requires epithelial folding events to shape the eye into a bi-layered optic cup that is initially asymmetric along the dorsal ventral axis (Casey et al., 2023). Whereas the dorsal and lateral aspects of the optic cup are roughly spherical, a fissure remains in the ventral portion, interrupting cup sphericity and accommodating the entrance of ocular vasculature and exit of retinal ganglion cell axons. Subsequent closure of this optic fissure involves numerous processes including tissue alignment, breakdown of the extracellular matrix, exclusion of intervening mesenchymal cells, and epithelial fusion (Patel and Sowden, 2019; Casey et al., 2021; Chan et al., 2021). When this process fails it results in an ocular defect known as a coloboma which can vary in its severity from benign structural changes to substantial visual impairment (ALSomiry et al., 2019; Lingam et al., 2021). Elucidating the developmental mechanisms governing optic fissure formation and closure is therefore critical to understand the etiology of this morphogenesis-based defect.

Optic fissure closure may be conceptually divided into two important steps: the approach of the optic fissure margins and the subsequent fusion process. Development of these margins can be first traced to the action of several signaling pathways that first pattern the early ventral optic vesicle and restrict the expression of a handful of key transcription factors to this region (Fuhrmann, 2010). Following these specification events, the margins morphologically appear simultaneously with optic vesicle involution generating a pocket for the invaginating lens pit that extends ventrally toward the optic stalk. As this involution occurs, a transitional zone containing wedge-shaped cells between the inner prospective neural retina (NR) and outer retinal pigmented epithelium (RPE) forms that is continuous with those of the developing optic fissure margins. This region harbors several adjacent apically constricted cells that are shaped by Rho-kinase signaling and actomyosin contractility priming further inward bending of the NR (Eiraku et al., 2011, 2012). Following formation of this zone, localized tissue expansion, basal constriction of NR cells, and flattening of the RPE continue to drive the formation of the bi-layered optic cup in organoids and embryos (Martinez-Morales et al., 2009; Eiraku et al., 2012; Nicolas-Perez et al., 2016; Oltean et al., 2016; Sidhaye and Norden, 2017; Moreno-Marmol et al., 2021). Whether the adoption of this shape is also driving the approach and alignment of the optic fissure margins during closure remains untested.

The fusion process facilitates the second step of closure once the optic margins align and contact each other. During this time, periocular mesenchymal tissue residing between the optic fissure margins is excluded, the basement membrane lining the basal side of the transitional zone breaks down, and cells from the opposing margins begin to intercalate with each other (Chan et al., 2020). These processes are necessary to generate a continuous layer of NR and RPE cells that are untethered from each other and requires the formation of junctions between new cell neighbors, the dissolution of others, and a breakdown in apical-basal polarity for a select number of cells (Gestri et al., 2018; Eckert et al., 2020). Apical-basal polarity must then be reestablished to ensure the continuity of a pseudostratified neural epithelium. The cellular mechanisms that drive epithelial intercalation are largely unexplored and new models are necessary to identify the contributions of specific events during this process.

Hints that cellular shape control may facilitate optic fissure closure comes from analysis of Shroom3 deficient embryos. Shroom3 is an actin- and Rho-kinase-binding molecule that facilitates the contraction of actomyosin within the apically positioned adherens junctional belt of epithelial cells (Hildebrand and Soriano, 1999; Hildebrand, 2005). Elevating the expression of Shroom3 in epithelial cells can induce apical constriction where it converts cells from a cylindrically shaped cell to a frustum or wedged shape (Hildebrand, 2005). This cell shape change is sufficient to drive the bending of epithelial tissues and in the absence of Shroom3, morphogenetic processes such as neural tube closure, lens and thyroid invagination, and gut rotation are disrupted (Hildebrand and Soriano, 1999; Nishimura and Takeichi, 2008; Chung et al., 2010; Plageman et al., 2010, 2011a). Shroom3 knock-out mice also appear to develop ventral optic cup abnormalities (Lang et al., 2014), but exactly how optic cup morphogenesis is disrupted in this model has yet to be investigated. Optogenetic activation of Shroom3 function in optic vesicle organoids can subtly alter their shape further supporting a role for Shroom3 during retinal morphogenesis (Martinez-Ara et al., 2022). Although Shroom3-mediated apical constriction is required and sufficient in certain contexts for tissue bending, the possibility remains that Shroom3 may facilitate additional processes occurring during morphogenesis. Indeed, compromised neural tube zippering, disorganized intestinal epithelia, distorted microtubule networks, and disrupted axonal architecture are all observed as a consequence of reduced Shroom3 function (Lee et al., 2007; Taylor et al., 2008; Grosse et al., 2011; Massarwa and Niswander, 2013). Given that morphogenetic mechanisms are typically multifactorial, it is useful to consider roles for Shroom3 that may be independent of apical constriction.

In this study, the function of Shroom3 was analyzed in the context of optic fissure closure in mouse embryos. Two distinct phenotypes that lead to colobomas are observed in Shroom3 mutants. In severely affected embryos the approach of the optic fissure margins is significantly attenuated. Although Rho-kinase-dependent apical constriction downstream of Shroom3 function is diminished, failure to bend the epithelium apically cannot explain the phenotype. Rather, a broader apical domain is present within the outer curvature, caused by altered cell shapes within the NR and RPE, which leads to the failure of optic margin approach. In both whole embryo and retinal specific Shroom3 knockout embryos, a novel type of coloboma was observed consisting of the formation of an ectopic infolding of the fused ventral NR. In these cases, fusion of the epithelial layers occurs normally, but the cells fail to reestablish apical-basal polarity uncovering a novel function for Shroom3 during epithelial fusion. Epithelial fusion of several additional developing tissues was also found to be Shroom3-dependent supporting a wider breadth of morphogenetic roles for Shroom3 during development.

## 2. Results

**Fig. 1.**
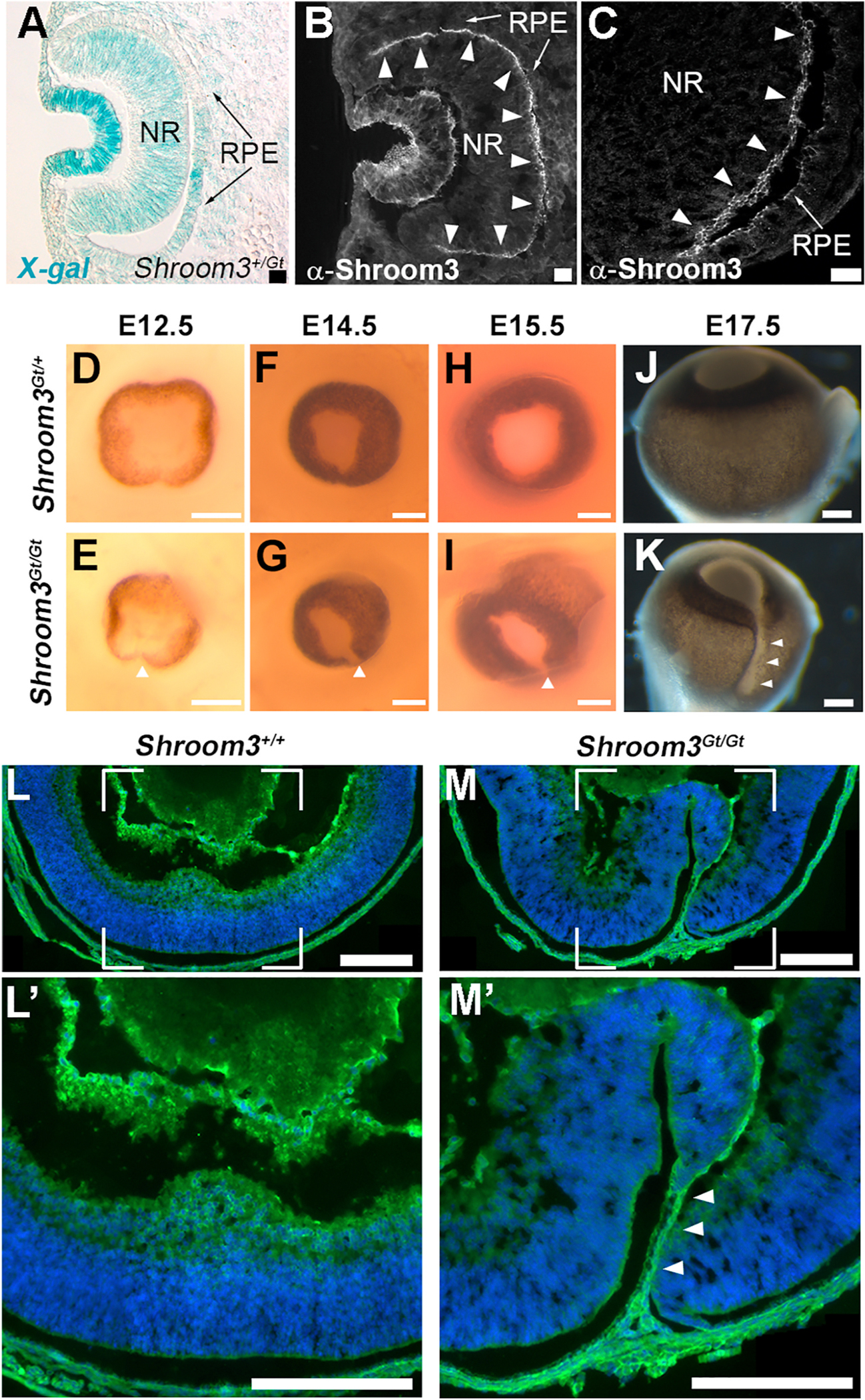
Shroom3 expression and localization in the mouse optic cup. A) Ocular expression of Shroom3 assessed via coronal cryosectioning and X-gal staining s of an E10.5 *Shroom3*^*Gt/*+^ mouse embryo. The gene-trapped allele drives β-galactosidase from the endogenous Shroom3 locus. B-C) Immunofluorescent staining of an E10.5 mouse embryo indicates apical localization of Shroom3 within both layers of the optic cup (OC) (arrowhead, neural retina; arrows retinal pigmented epithelium). D-I) *En face* view of control (D, F, H) and mutant (E, G, I) embryonic eyes at the indicated stage. J-K) Ventral view of whole eyes extracted from E17.5 mouse embryos. Note the presence of a gap in pigment in mutant embryos (n = 10/12, 83.3 %), compared with control embryos (n = 0/7) implicating a lack of ventral fusion (arrowheads E, G, I, K). Sagittal cryosections of E17.5 mouse eyes immunostained for laminin (green) and nuclei (blue). The squared areas are magnified and depicted in the bottom panels and arrowheads mark a thin layer of mesenchymal tissue between the optic cup margins. Scale bars: A-C, 20 μm; D-M, 200 μm. (For interpretation of the references to colour in this figure legend, the reader is referred to the Web version of this article.)

A disruption to a number of distinct cellular behaviors during optic cup morphogenesis such as epithelial folding, cell shape changes, altered proliferation, basement membrane regulation, and cell adhesion, have all been implicated as an underlying cause of ocular coloboma (Patel and Sowden, 2019; Chan et al., 2020). We previously observed ventral optic cup abnormalities in mouse embryos deficient for Shroom3 (Plageman et al., 2014). To further investigate the role of Shroom3 during this process we first determined whether it is expressed during optic cup morphogenesis. Analysis of cryosectioned, X-gal stained *Shroom3*^+*/Gt*^ E10.5 embryos, which harbor a gene-trapped β-galactosidase/cre-recombinase coding sequence, was performed to observe which cells express Shroom3 (Fig. 1A) (Hildebrand and Soriano, 1999). While stronger expression is observed in the lens, X-gal signal in both epithelial layers of the optic cup is also apparent with some variable staining observed in the presumptive RPE (Fig. 1A). Antibody staining at the same stage reveals that Shroom3 is apically localized in the presumptive neural retina (arrowheads Fig. 1B–C) and retinal pigmented epithelium (arrow, Fig. 1B–C), similar to observations made in other epithelia (Hildebrand, 2005; Plageman et al., 2010, 2011a; Loebel et al., 2016; Lawlor et al., 2023). The coloboma phenotype of homozygous *Shroom3*^*Gt/Gt*^ embryos is first obvious in whole eyes at E12.5 as a sharper inward bend and gap in pigmented tissue (Fig. 1D vs. 1E). This gap in pigment is observed in eyes for the duration of embryonic development (Fig. 1F–K, arrowheads). To ascertain if this gap was indicative of a coloboma, laminin localization at E17.5 was observed by immunofluorescently labeled sections. The presence of a coloboma was confirmed by the presence of a thin layer of mesenchymal-like tissue remaining between the margins of the optic cup in mutant embryos (n = 10/12, 83.3 %) arrowheads, Fig. 1M) while fusion completed normally in control embryos (n = 7/7, 100 % Fig. 1L). The absence of epithelial fusion by this late developmental stage indicates that the process is not simply delayed and is representative of a a typical coloboma phenotype.

**Fig. 2.**
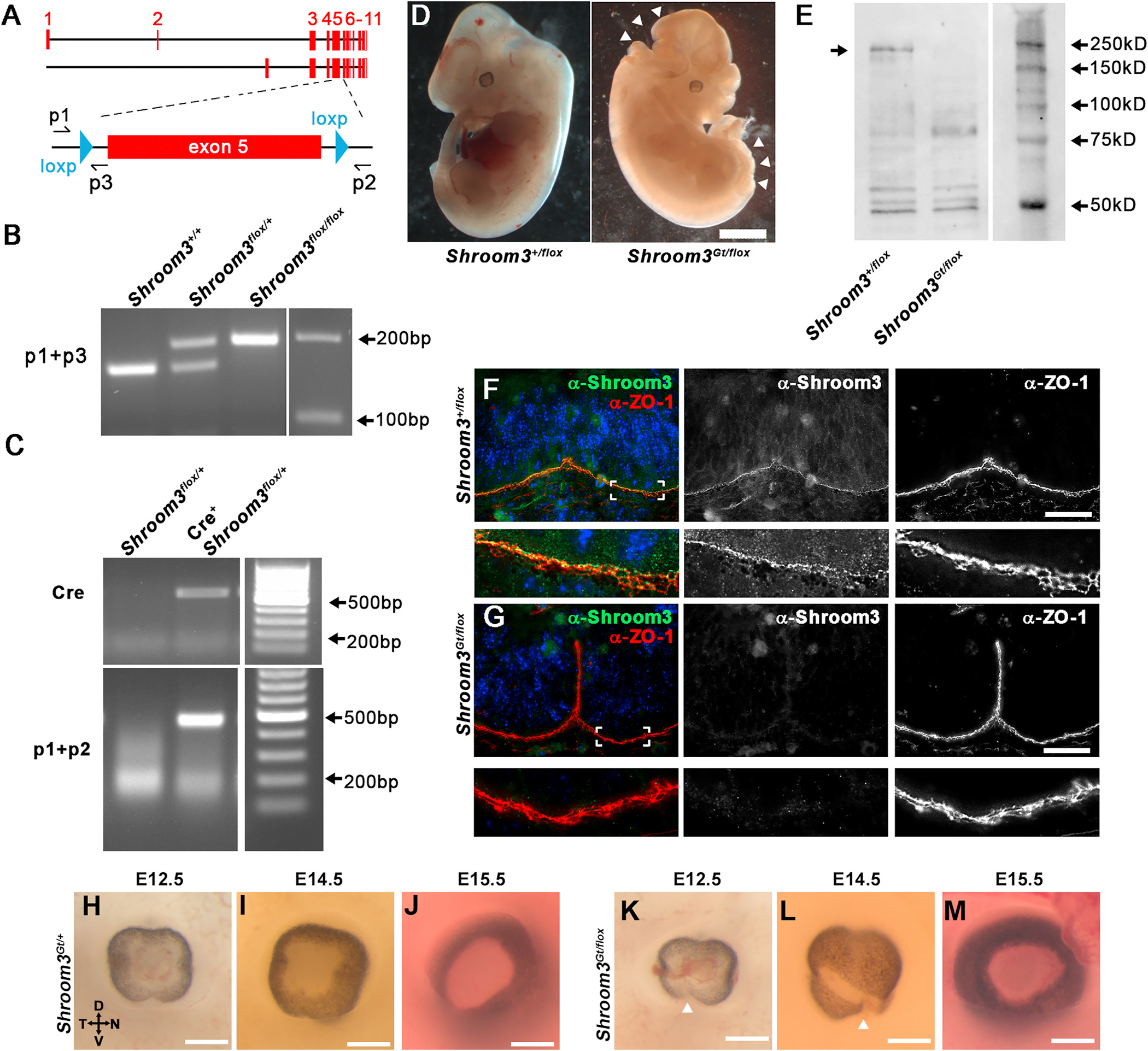
Generation and validation of a Shroom3 conditional allele. A) Exon diagram of the long (top line) and short (bottom line) isoforms of mouse Shroom3. The region near exons 5 and 6 are magnified and the location of the loxp sites and validating genomic primer sequences are indicated. Note that the conditional allele should affect both Shroom3 isoforms. B) Genomic PCR of mice harboring 0, 1, or 2 alleles of the floxed gene. C) Genomic PCR of retinal tissue following cre mediated recombination of Shroom3. Note that the presence of the ~500bp band indicates that recombination has occurred. D) Whole E12.5 embryos with the indicated genotype. Note that the neural tube defects exencephaly and spina bifida are observed in *Shroom3*^*Gt/flox*^ embryos. Because the *Shroom3*^*Gt*^ allele drives expression of cre-recombinase everywhere Shroom3 is normally expressed, recombination of the *Shroom3*^*flox*^ allele should occur in all of these tissues and effectively mimicking the germline KO phenotype. E) Western blot of protein lysates derived from the indicated embryos utilizing a C-terminal Shroom3 targeted antibody. Note the loss of a ~220 KD band that corresponds to the size of the full length of Shroom3 protein. F-G) The ventral optic cup of E12.5 mouse embryos of the indicated genotype were sagittally cryosectioned and immunofluorescently co-labeled using a Shroom3 and ZO-1 antibody. Shroom3 labeling is absent from the apical junctions of the neural retina and RPE of mutant embryos. H-M) Whole embryonic eyes of the indicated stages were viewed *en face* from control (H–J) and Shroom3 mutant (K–M) embryos. There is a disruption in the continuous ventral RPE pigment of some (K-L, arrowheads) but not all (M) mutant eyes.

**Fig. 3.**
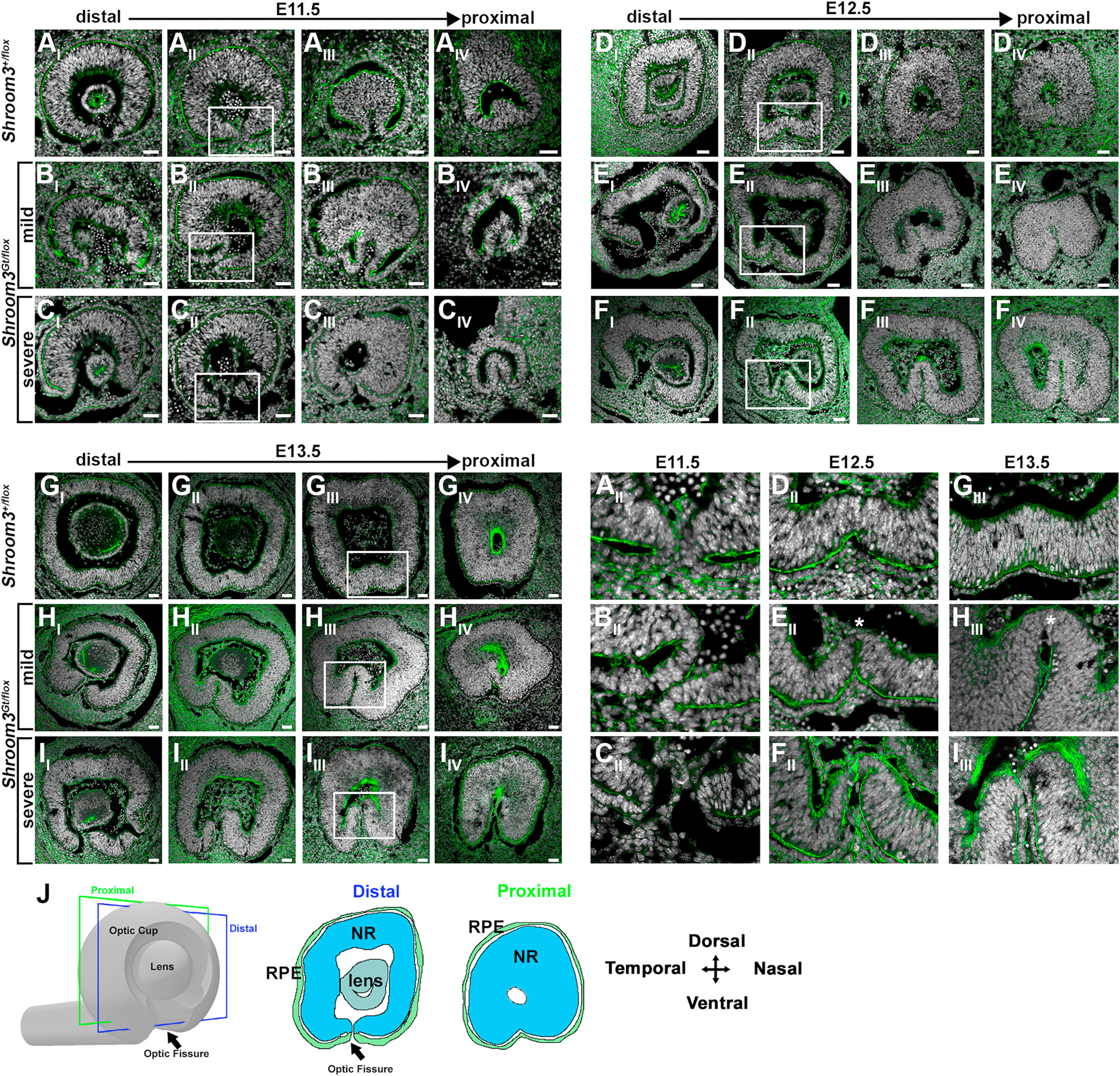
Optic fissure closure abnormalities in Shroom3 deficient embryos. F-actin (green) and Hoechst (white) stained sagittal cryosections of mouse optic cups at E11.5 (A–C), E12.5 (D–F), and E13.5 (G–I). Representative images from a single eye of control (A, D, G), mildly affected (B, E, H), and severely affected embryos (C, F, I) along the proximal-distal axis relative to the brain are depicted. The white rectangle indicates an optic cup region immediately behind the lens which are magnified and displayed in the bottom right quadrant. The white asterisks represent a thin epithelium that maintains a connection of the neural retina in mildly affected embryos. J) The diagrams indicate the relative plane of section along the proximal distal axis during immediately preceding epithelial fusion and the location of the lens, neural retina (NR), and retinal pigmented epithelium (RPE). (For interpretation of the references to colour in this figure legend, the reader is referred to the Web version of this article.)

In order to determine how Shroom3 deficiency leads to a coloboma a conditional knock out allele of *Shroom3* was generated to permit a tissue specific approach. The targeting vector was designed such that exon 5 of the *Shroom3* gene was flanked with loxP sites and upon recombination would cause the generation of a stop codon to be placed in frame, prematurely terminating translation of the protein prior to the actin- and rho-kinase-binding domains (Fig. 2A). Genomic PCR confirmed that the exons were properly flanked (Fig. 2B) and that recombination occurs in the presence of a *cre* transgene (Fig. 2C). Loss of Shroom3 function/protein in this model is best demonstrated by the presence of an exencephalic phenotype observed in 100 % (37/37) of experimental embryos that harbor one copy of the floxed allele and one copy of the gene-trapped allele (*Shroom3*^*Gt/flox*^) (Fig. 2D). This stands in contrast to 0 % (61/61) of control embryos that harbor a single flox and wild type allele (*Shroom3*^+*/flox*^) (Fig. 2D.) or a single gene-trapped and wild-type allele (*Shroom3*^+*/Gt*^) as previously observed (Plageman et al., 2014). Western blotting of protein lysates from *Shroom3*^+*/flox*^ and *Shroom3*^*Gt/flox*^ embryos reveals loss of protein expression in mutant embryos (Fig. 2E). The loss of Shroom3 protein is confirmed from immunofluorescently labeling E12.5 cryosections *Shroom3*^+*/flox*^ and *Shroom3*^*Gt/flox*^ embryos with a Shroom3-specific antibody (Fig. 2F vs 2G). Once established as a functional conditional allele, the eyes from each genotype were analyzed during and after the timeframe of optic fissure closure. Observing whole eyes from E12.5-E15.5 revealed that while control embryos maintained a continuous layer of ventral pigmented tissue following fusion 12/12, 100 %) (Fig. 2H–J), a subset of *Shroom3*^*Gt/flox*^ mutant embryos possessed a clear coloboma phenotype with a ventral gap in pigment (9/20; 45.0 %) (Fig. 2K–L, arrowheads). When the gap in pigment was absent the ventral optic cup often maintained an exaggerated ventral v-shaped indentation (Fig. 2L) or looked indistinguishable from control embryos of the same age (11/20; 55.0 %) (Fig. 2J vs. 2M). To determine if epithelial fusion was occurring normally, sagittal cryosections through the optic cup were prepared and fluorescently labeled with phalloidin to observe morphology. At E11.5, distal sagittal cryosections of control and mutant embryos revealed the presence of blood vascular tissue between the ventral optic cup margins that appear to maintain their separation, although the margins of mutant embryos appear further apart than controls (Fig. 3A_I_, vs. 3B_I_, and 3C_I_). More proximally, the margins in control and mildly affected mutants approach each other and make contact, which presumably enables fusion to occur (Fig. 3A_II-IV_, B_II-IV_). But when this contact is made in the mildly affected embryos the margins appear unaligned (Fig. 3B_II_). This phenotype can be distinguished from those that appear more severely affected, and the margins of the ventral optic cup often do not contact each other, remaining separated along the entire proximal-distal axis (Fig. 3C_I-IV_). The lack of fusion is maintained in the severely affected eyes through E13.5 in contrast to control eyes which always fuse (10/10, 100 %) (Fig. 3I, compared to 3G). However, mildly affected embryos that do permit fusion develop a unique phenotype where an ectopic ventral bend is observed beginning at E12.5 (Fig. 3E and H). A similar ventral bend normally appears in wild-type embryos (Bernstein et al., 2018), however it is typically resolved by E13.5 (Fig. 3D and G). In contrast, some mutant embryos develop a more pronounced ventral bend with variable severity (Fig. 3E vs. Fig. 3F). Categorizing the mutants based on the success (mild) or failure (severe) of ventral optic margin approach and the presence (mild) or absence (severe) of epithelial fusion led to the observation that the mild phenotype occurred in 7/16 (43.8 %) mutant embryos while the severe phenotype occurred in 9/16 (56.3 %) of mutant embryos.

**Fig. 4.**
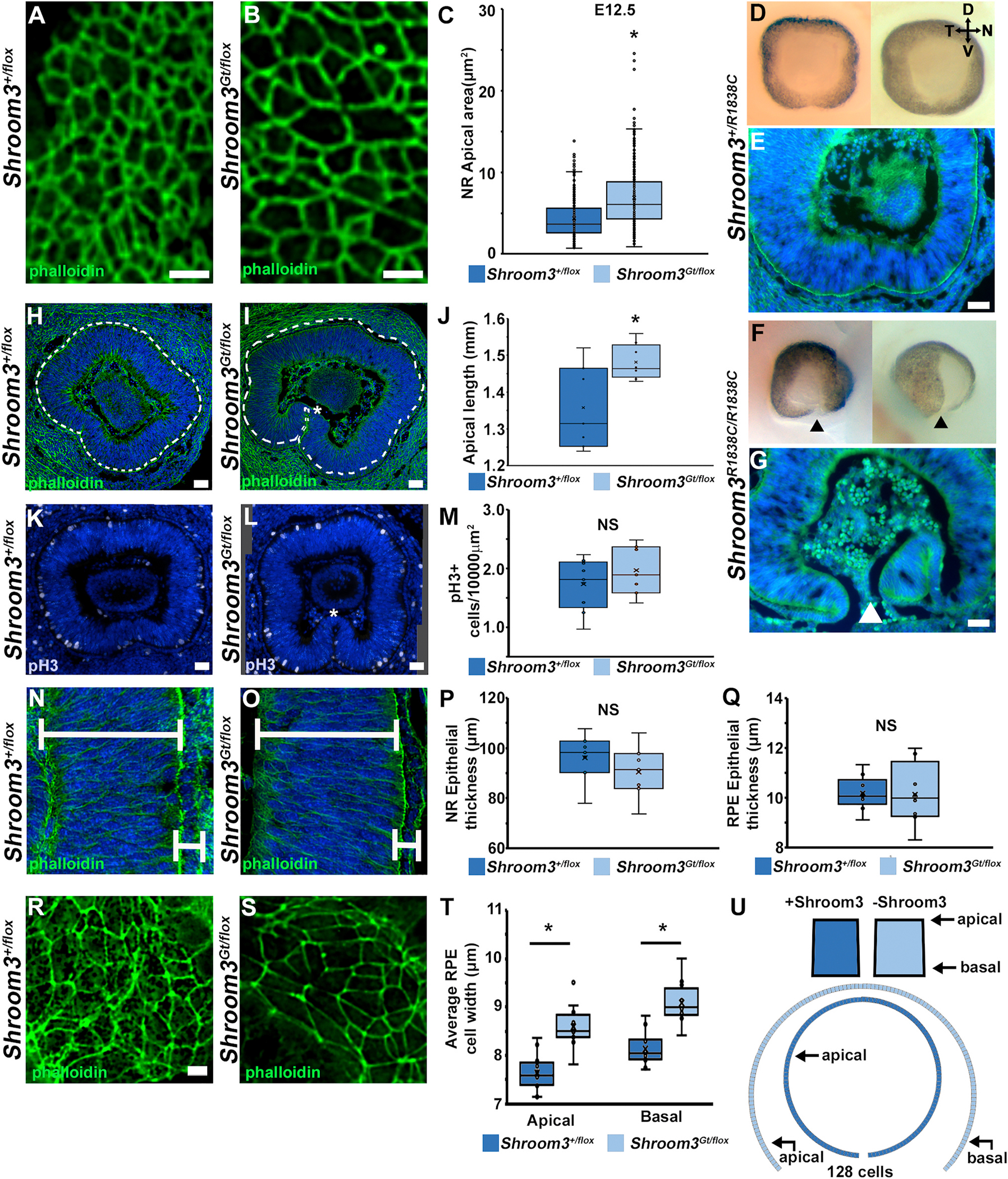
Loss of Shroom3 causes an expansion of the apical surface area of the optic cup. A-B) *en face* view of the apical face of control and mutant E12.5 neural retinas stained with phalloidin. C) Results of the quantitative analysis of individual cell apical areas from one eye of 6 different embryos of each genotype are represented as box plots with an exclusive median. Note that a significant difference is observed between genotypes (p < 0.01). D-G) Whole eyes from heterozygous (D) and homozygous (F) E12.5 embryos with 1 or 2 copies of the Rho-kinase binding mutant of Shroom3. Sagittal sections of E12.5 optic cups immunolabeled with phalloidin (green) and Hoechst (blue) indicate that homozygous mutants (G) maintain an open optic fissure and the margins do not approach each other while the heterozygous embryos undergo closure normally (E). H-I) Selected images of sagittal sections at the level of the posterior lens of E12.5 optic cups immunolabeled with phalloidin (green) and Hoechst (blue) with the indicated genotypes. The length of the apical side of the neural retinal epithelium was traced with a white line. J) The length of the lines represented in (H–I) were measured from at least 7 different control and mutant embryos and represented as box plots with an exclusive median. Note that mutant embryos had significantly more apical surface area (p *<* 0.05). K-L) Selected images of sagittal sections at the level of the posterior lens of E12.5 optic cups immunolabeled with the cell proliferation marker pH3 (magenta) and Hoechst (blue) with the indicated genotypes. M) The number of proliferative cells per unit area in (K–L) were counted from at least 7 different control and mutant eyes and represented as box plots with an exclusive median. No significant differences are observed between phenotypes. N-O) Selected images of the lateral neural retina epithelium from E12.5 sagittal sections fluorescently labeled with phalloidin (green) and Hoechst (blue). The white bars represent the apical-basal length of the NR and RPE epithelia. P-Q) The thickness of the NR (P) and RPE (Q) epithelia from each genotype was measured from 4 to 7 different embryonic eyes at E12.5 and the results represented as box plots with an exclusive median. No significant differences in thickness were observed. R-S) *en face* view of the apical face of control and mutant E12.5 RPE cells stained with phalloidin. T) The average apical and basal widths of RPE cells from four different embryos were quantified by calculating the quotient of the number of nuclei in a mid-optic cup sagittal section of the entire eye and the length along the entire apical or basal side of this epithelial layer and depicted on the graph. Note that RPE cells are significantly wider apically and basally in the absence of Shroom3 (p *<* 0.01). U) The average trapezoidal shape of RPE cells from each genotype from the average dimensions from Q, T is depicted. A simple 2d model of these shapes were generated from laterally aligning 128 cells recreating what may occur in the outer curvature of the retina. Note the distinct difference in the curvature of the model epithelia between genotypes Scale bars A-B, R–S: 5 μm; E-G, H-I, K-L, N–O: 50 μm). (For interpretation of the references to colour in this figure legend, the reader is referred to the Web version of this article.)

Shroom3 functions to induce the contraction of the actomyosin associated with the apically positioned adherens junctional belt that can often lead to apical constriction of an epithelial cell. To determine if the abnormal optic cup bending could be due to a reduction in apical constriction, the apical areas of optic cup/neural retinal cells were observed from phalloidin (F-actin) labeled optic cups (Fig. 4A–B) and measured from tangential cryosections of control and mutant animals (Fig. 4C). Sectioning in this manner allows the apical junctions to be parallel with the slide enabling accurate size measurements. *Shroom3*^+*/flox*^ optic cup cells normally reduce their mean apical areas during the fusion process from E11.5 (8.2 μm^2^) to E12.5 (4.4 μm^2^). While a reduction in apical areas also occurs in *Shroom3*^*Gt/flox*^ mutant embryos from E11.5 (13.6 μm^2^) to E12.5 (6.7 μm^2^), these means are significantly increased between genotypes (T-test: E11.5, p = 2.2 × 10^−19^; E12.5, p = 8.6 × 10^−29^) (Fig. 4C–D). This phenotype is Rho-kinase dependent as mice with a homozygous missense mutation in the domain of Shroom3 that associates with Rho-kinase also have colobomas where the optic margins fail to approach each other (Fig. 4D–G) (Marean et al., 2011). Apical constriction is commonly thought to induce the bending of an epithelial tissue such that the apical side resides on the inner curvature. In contrast, the apical side of the neural retinal epithelium of the optic cup is positioned within the outer curvature rather than the inner curvature. Thus, it is likely insufficient to explain the mutant phenotype as a failure of apical constriction-induced epithelial bending. To investigate the possibility that the reduction of apical constriction increases the surface area of the outer curvature, the two-dimensional length of the apical side of the epithelium in sagittal cross-sections through control and mutant E12.5 optic cups at the level of the lens where the failure of normal fusion can be observed was performed (Fig. 4H–I). The mean apical length from *Shroom3*^+*/flox*^ optic cups was 1.38 mm, while the mean apical length *Shroom3*^*Gt/flox*^ mutant optic cups was 1.48 mm (T-test: p = 0.014). This consistent increase in length suggests that the surface area is indeed greater along the apical curvature in mutant embryos. To rule out the possibility that this is caused by an increase in cell proliferation, cryosections were immunolabeled with a phospho-histone marker (Fig. 4K–M) The mean number of pH3 positive cells per unit area did not significantly change in the absence of Shroom3 (Figs. 4M and 2.24 vs. 2.47 pH3+ cells/10000 μm^2^; T-test: p = 0.29) indicating that the surface area increase is not due to a rise in proliferative index in the absence of Shroom3. Because epithelial length is also affected by Shroom3 in a number of contexts, including optic vesicle organoids (Lee et al., 2007; Grosse et al., 2011; Plageman et al., 2011a; Martinez-Ara et al., 2022), the length of the NR cells were measured. Surprisingly, dorsal/lateral wall thickness of the optic cups were unchanged (Fig. 4N–P). However, a wide variety of neuroepithelial thicknesses were observed at the ventral fusion point in the mildly affected mutants where the epithelium often remains very thin (Fig. 3E, H, 4I, 4L; asterisks). Because Shroom3 is removed from all cells in these mutants we also determined the average geometry of RPE cells. Cell heights of RPE cells are not significantly different between genotypes (Fig. 4Q, T-test: p = 0.63). The apical junctional areas when imaging *en face* patches of RPE cells appear larger in the mutant vs. control (Fig. 4R–S) but we found this to be highly variable depending on the location. This is especially true for the dorsal vs. ventral RPE cells where the dorsal cells tend to be relatively thin and wide while ventral cells tend to be taller and thinner. To counter this, we analyzed sagittal sections through the middle of the lens within the optic cup and counted the cells along the RPE perimeter and measured the linear apical and basal lengths of the epithelium (similar to panels 4H and I). The average apical and basal length per cell was calculated for 3 sections from four different embryos (12 sections/genotype) and depicted on a graph (Fig. 4T). RPE cells on average were wider both apically (7.6 μm vs. 8.6 μm, T-test: p = 7.78 × 10^−6^) and basally (8.1 μm vs. 9.1 μm, T-test: p = 3.11 × 10^−3^) in the mutants suggesting that the absence of Shroom3 causes these cells to increase both their apical and basal areas. As a way to determine if this change in geometry could also affect optic cup morphogenesis, a simple model was generated by utilizing the average trapezoidal shape of RPE cells calculated from the data in Fig. 4Q and T for each genotype (Fig. 4U, top) and aligning their lateral sides. We aligned 128 model cells and found that the somewhat subtle change in geometry of individual cells can greatly impact how an epithelium could be shaped (4U, bottom). Control cells almost form a circle from 128 model cells, while the same number of mutant cells form more of a crescent shape. These distinct model epithelia closely match the geometry of control and severely affected Shroom3 mutant embryos with a coloboma and suggest that the change in RPE geometry contributes to the inability of the optic margins to align themselves during morphogenesis.

**Fig. 5.**
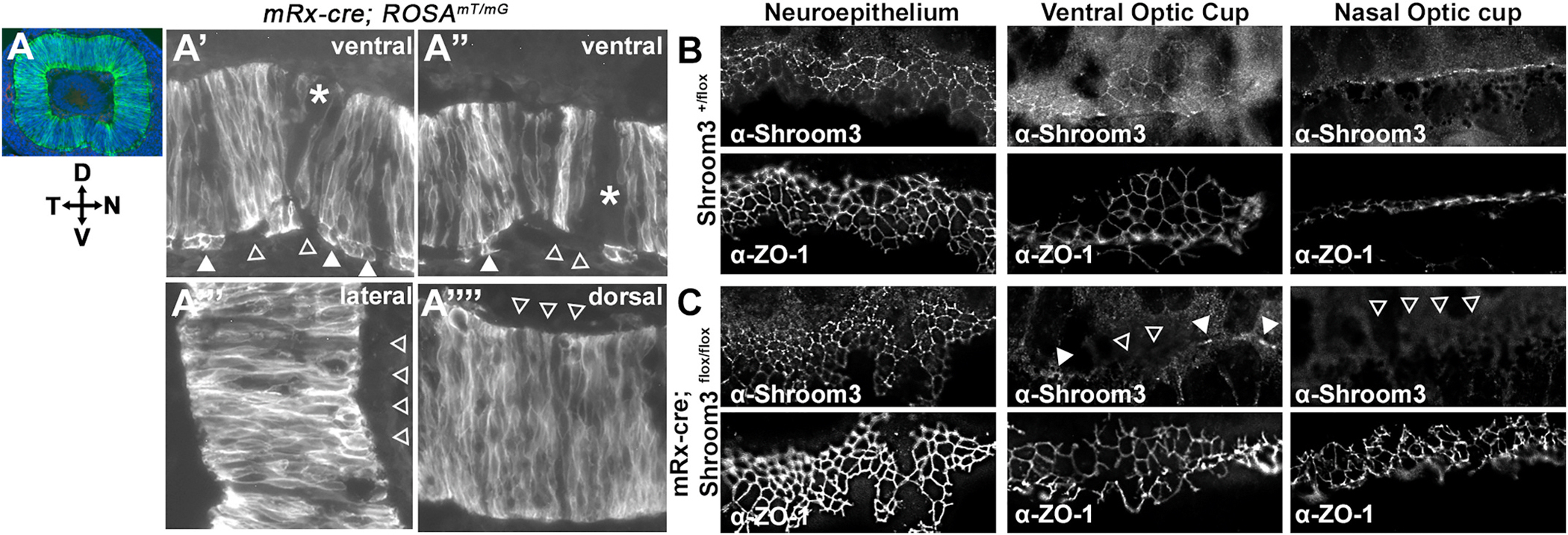
Shroom3 is mostly absent from the optic cup of cKO mutant embryos. A) Image of a sagittally sectioned E12.5 embryos derived from a cross with a cre-sensitive reporter line. Green/white signal indicates cells that have undergone recombination and express GFP. Note that cre activity is present in most neural retinal cells and mostly absent from the RPE and other ocular tissues such as the lens and periocular mesenchyme. Magnified images of distinct regions within the optic cupthe are depicted in the right panels. Solid and open arrowheads indicate cre+ and cre− RPE cells, respectively. The asterisk indicates NR cells that do not express GFP. B-C) Immunofluorescent labeling performed on sagittal cryosections through the neuroepithelium and optic cups of control and cKO mutant embryos at E12.5 using antibodies specific for Shroom3 and ZO-1. Apical junctional localization is apparent for both Shroom3 and ZO-1 in all epithelia examined of control embryos (B) but is absent from most of the cells in the ventral optic cup (C, middle, open arrowheads) and completely absent from the nasal optic cup (C, far right, open arrowheads). A few cells appear to possess apical Shroom3 in the ventral optic cup (C, middle, closed arrowheads). Scale bar = 5 μm). (For interpretation of the references to colour in this figure legend, the reader is referred to the Web version of this article.)

**Fig. 6.**
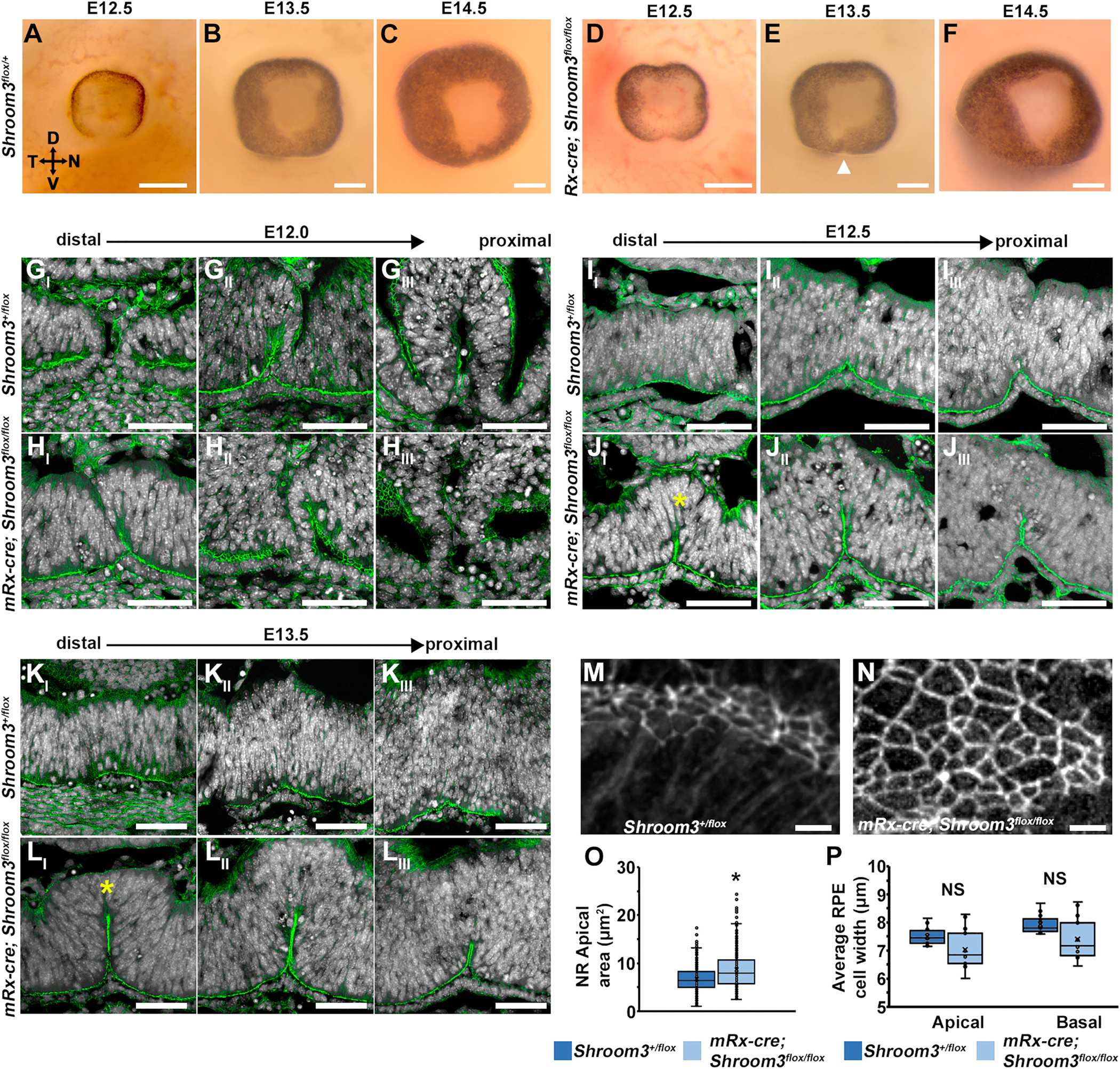
Shroom3 is required within the neural retina during optic fissure closure. A-F) Whole embryonic eyes viewed *en face* from control and conditionally mutant embryos at the indicated stages. Note that conditional ablation of Shroom3 typically does not cause a gap in ventral pigment. G-L) F-actin (green) and Hoechst (white) stained sagittal cryosections of mouse optic cups at E12.0 (G–H), E12.5 (I–J), and E13.5 (K–L). Representative images from a single eye of control embryos (G, I, K) and embryos with a neural retina specific deletion of Shroom3 (H, J, L) along the proximal-distal axis relative to the brain are depicted. The asterisks mark the thin ventral epithelium at the fusion point in Shroom3 mutant eyes. M-N) Tangential sections through the apical junctions of NR cells from control and Shroom3 mutants were fluorescently labeled with phalloidin. O) Results of the quantitative analysis of individual cell apical areas from 3 different embryonic eyes from each genotype are represented as box plots with an exclusive median. Note that a significant difference is observed between genotypes (p *<* 0.01). P) The average apical and basal widths of RPE cells from three different embryos were quantified by calculating the quotient of the number of nuclei in a sagittal section and the linear apical or basal length and depicted on the graph. No significant difference was calculated. Scale bars A–L: 50 μm; M–N: 5 μm). (For interpretation of the references to colour in this figure legend, the reader is referred to the Web version of this article.)

In order to define the contribution of Shroom3 within the NR vs. the surrounding mesenchymal tissues, the nearby lens, or the RPE, an optic cup specific cre (mRx-cre) was utilized to conditionally ablate *Shroom3* (Klimova et al., 2013). To first determine how well this cre functions in the ventral retina during fusion, a GFP reporter line was utilized to genetically mark the affected cells. It was observed that cre activity of the mRx driver is found in most of the neural retinal cells, but only some of the RPE cells (Fig. 5A, closed vs. open arrowheads). A few cells within the NR near the fusion point were also missing GFP (Fig. 5A, asterisks). Mouse embryos with the genotype *mRx-cre*; *Shroom3*^*flox/flox*^ (hereafter, Shroom3 cKO) were then analyzed for Shroom3 and ZO-1 expression via immunofluorescent staining in the neuroepithelium where cre is not expressed as a control (Fig. 5B, left), in the ventral (Fig. 5B middle) and nasal optic cup (Fig. 5C, right). In control eyes, Shroom3 is abundant in the apical junctions of neuroepithelial and optic cup tissue in control embryos. The protein is absent from most of the optic cup (Fig. 5C, open arrowheads) however small patches of Shroom3 positive apical junctions (Fig. 5C closed arrowheads) are detected in the ventral region where the GFP reporter signal was notably absent. The Shroom3 cKO eyes typically do not have a separation in the pigment near the ventral optic fissure closure point and fusion looks relatively normal (n = 16) when observed *in situ* from E12.5-E14.5 (Fig. 6A–F). Although an exaggerated ventral cleft in the pigment was sometimes observed in the mutant (n = 6/16, Fig. 6E, arrowhead) but not in control embryos (0/17). However, histological sectioning reveals that while fusion of the presumptive RPE occurs normally, the ectopic ventral bend observed in the mildly affected *Shroom3*^*Gt/flox*^ mutant embryos are also present in the Shroom3 cKO mutants (Fig. 6G–L). Fusion appears to begin normally at E12.0 control and mutant embryos (Fig. 6G vs. 6H). However, while the presumptive RPE regains its flat monolayer morphology at E12.5, the neural retina fusion point remains relatively thin and bent inward in Shroom3 cKO mutants (n = 8/10; Fig. 6J and L) while control embryos possess a smaller inward bend that eventually resolves, (n = 14/14; Fig. 6I and K). The absence of Shroom3 causes an increase the apical areas of presumptive neural retinal cells in Shroom3 cKO eyes (Fig. 6M–O, 6.7 μm^2^ vs. 8.7 μm^2^, T-test: p = 3.78 × 10^−18^). In contrast, the average apical and basal widths of RPE cells are not significantly changed in the Shroom3 cKO mutant eyes (Fig. 6P, T-test: p = 0.25 and p = 0.27, respectively). The lack of an RPE phenotype and the retained ability of the optic margins to approach and fuse in the cKO mutants further supports a direct role for Shroom3-regulated geometry of RPE cells during normal ocular morphogenesis.

**Fig. 7.**
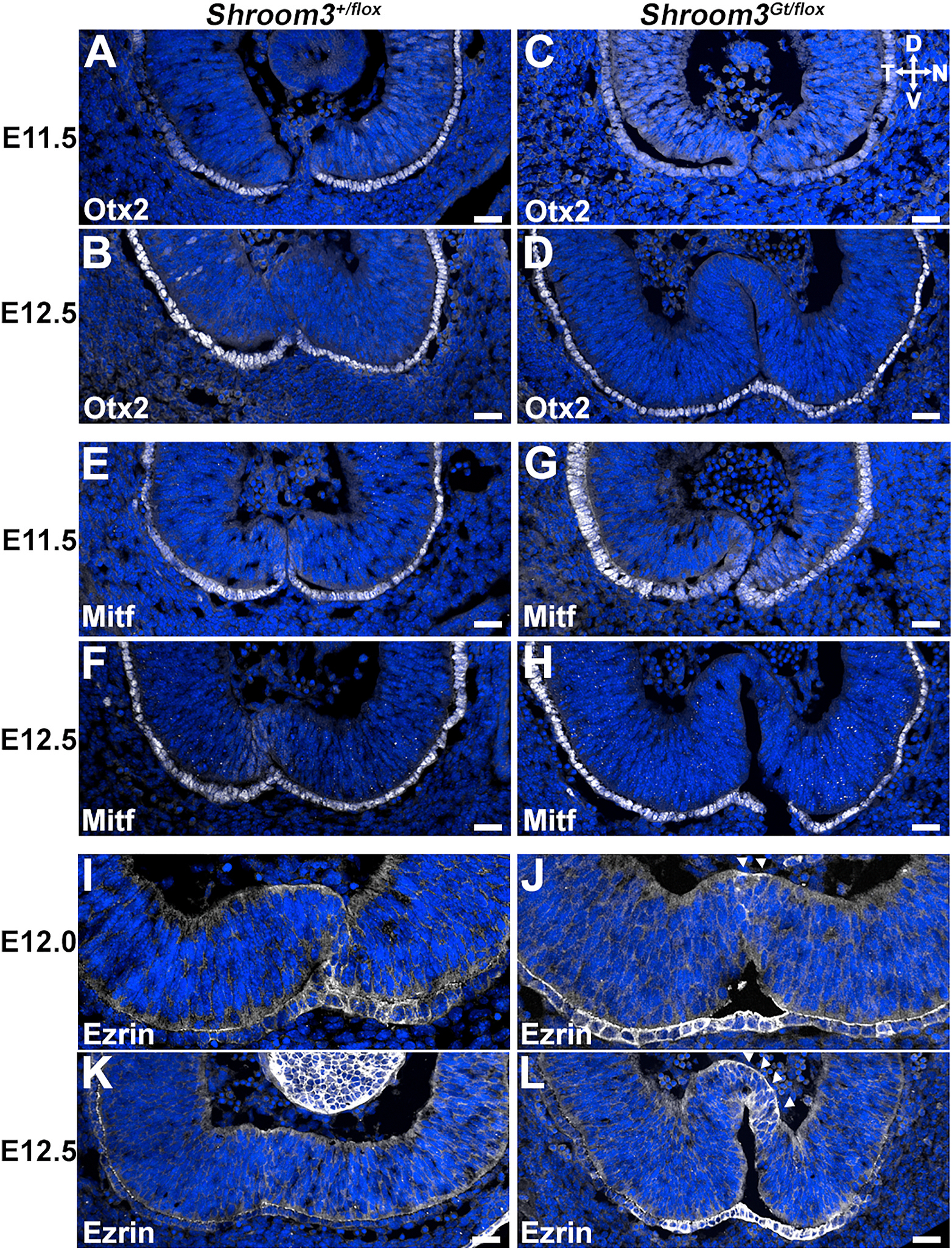
RPE cell specification is unaffected in Shroom3 mutants. Ventral views of sagittal sections of embryonic eyes from control and Shroom3 mutant embryos at the indicated ages were immunofluorescently labeled for the RPE markers Otx2 (A–D), Mitf (E–H), and Ezrin (I–L) (white) and Hoechst (blue). Note that the thin epithelium at the fusion point in mutant embryos lacks expression of Otx2 and Mitf indicating that the thickness of the tissue is likely not due to aberrant RPE specification. Also note the presence of the cytoskeletal protein Ezrin (arrowheads) in this region suggesting that although not specified as RPE the tissue displays signs of cytoskeletal disruptions. (For interpretation of the references to colour in this figure legend, the reader is referred to the Web version of this article.)

**Fig. 8.**
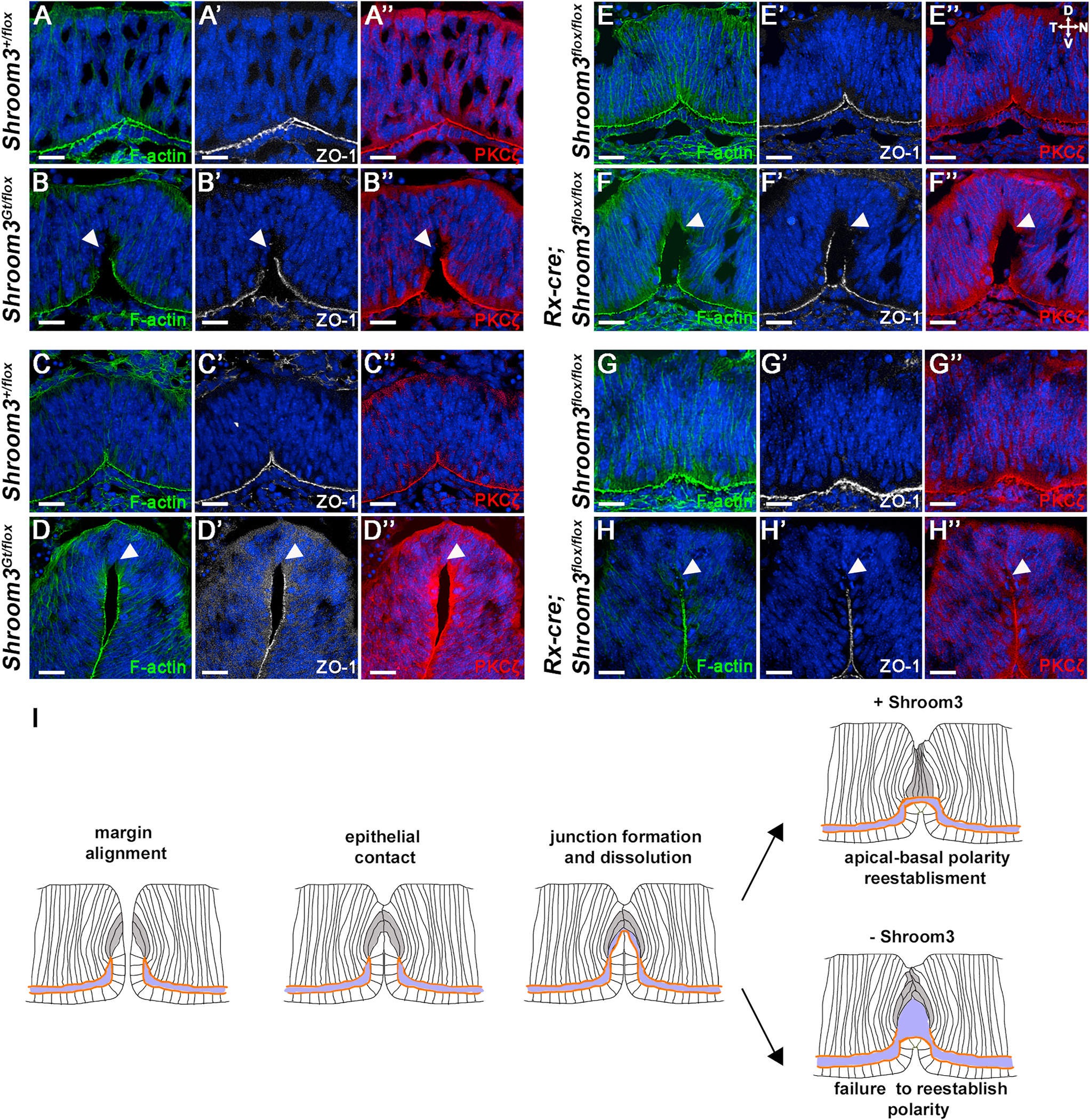
Apical markers are discontinuous at the fusion point in Shroom3 mutant embryos. A-L) Ventral views of the fusion point from E12.5 (A–D) and E13.5 (E–H) sagittal sections of embryonic eyes from control (A,C,E,G) and Shroom3 mutant embryos (B,D,F,H) immunofluorescently stained with phalloidin (green), ZO-1 (white), or PKCζ (red) and Hoechst (blue). Arrowheads mark locations of the fusion point apex with an absence of the indicated apical markers. I) Model of how the absence of Shroom3 affects the fusion process of ventral optic cup margins from ~E11.5-E12.5. The same grey cells are traced at each stage and the red line represents the apical face of the neural retina and the blue represents the space between the RPE and neural retina. While the initial stages of contact, junction formation between new neighboring cell populations, and separation of the neural retina from the RPE, occur normally, the grey cells are thought to normally lost apical-basal polarity but then reestablish this polarity as fusion concludes. However, due to the presence of cells without apical markers, it is hypothesized that these cells fail to reestablish polarity and prevents normal tissue thickening, causing the epithelium to remain thin and maintain an ectopic ventral bend. Scale bars: 20 μm. (For interpretation of the references to colour in this figure legend, the reader is referred to the Web version of this article.)

The presence of the ectopic ventral bend prompted further investigation into the role of Shroom3 during fusion. To test the hypothesis that the thin epithelium is due to RPE-like cells trapped in the neural retinal layer, cryosections were immunolabeled for the RPE fate markers Otx2 and Mitf. Neither transcription factor appeared present in the neural retinal tissue of control or *Shroom3*^*Gt/flox*^ mutant embryos at E11.5 or E12.5 but are strong in the presumptive RPE suggesting that although thin, the cells in this involuted region are likely specified correctly (Fig. 7A–H). The cytoskeletal protein Ezrin, which is also expressed in the RPE (Bonilha et al., 1999, 2006) does appear to be mislocalized. At the early stages of fusion, E12.0 embryos normally have some elevated Ezrin staining at the fusion point (Fig. 7I and J). But in *Shroom3*^*Gt/flox*^ mutant embryos this localization persists within the thinner neural epithelium while it disappears from the fusion point of control embryos (Fig. 7K vs. 7L). Together this data suggests that although cell fate may be unchanged, the cytoskeleton of these cells is disturbed. Because Ezrin is also known as a marker of the apical plasma membrane (Sauvanet et al., 2015), it was surmised that apical polarity may be disturbed. Indeed, Ezrin localization in the ectopic region appears localized in the basal and lateral plasma membrane of the *Shroom3*^*Gt/flox*^ mutant embryos (Fig. 7J–L, arrowheads). Analysis of the apically positioned F-actin belt using phalloidin staining reveals a continuous apical signal at the fusion point in control embryos at E12.5 (Fig. 8A) and E13.5 (Fig. 8C). However, it was often observed that apical phalloidin staining along the proximal distal axis of the fusion point in *Shroom3*^*Gt/flox*^ mutant embryos was discontinuous at both stages (n = 6) (Fig. 8B and D, arrowheads). Likewise, the tight junctional marker ZO-1 and the Par polarity complex marker PKCζ were continuous in the control embryos (Fig. 8A,C) but missing in the same region of the *Shroom3*^*Gt/flox*^ mutant embryos (Fig. 8B–D, arrowheads). The discontinuous localization of polarity markers suggests that there is a subset of cells at this apex that are not correctly polarized. Similar results were observed when analyzing the retinal specific cKO mutants and the apical polarity markers remained continuous in control embryos (Fig. 8E–G) but discontinuous in the mutant embryos (Fig. 8F–H, arrowheads)

**Fig. 9.**
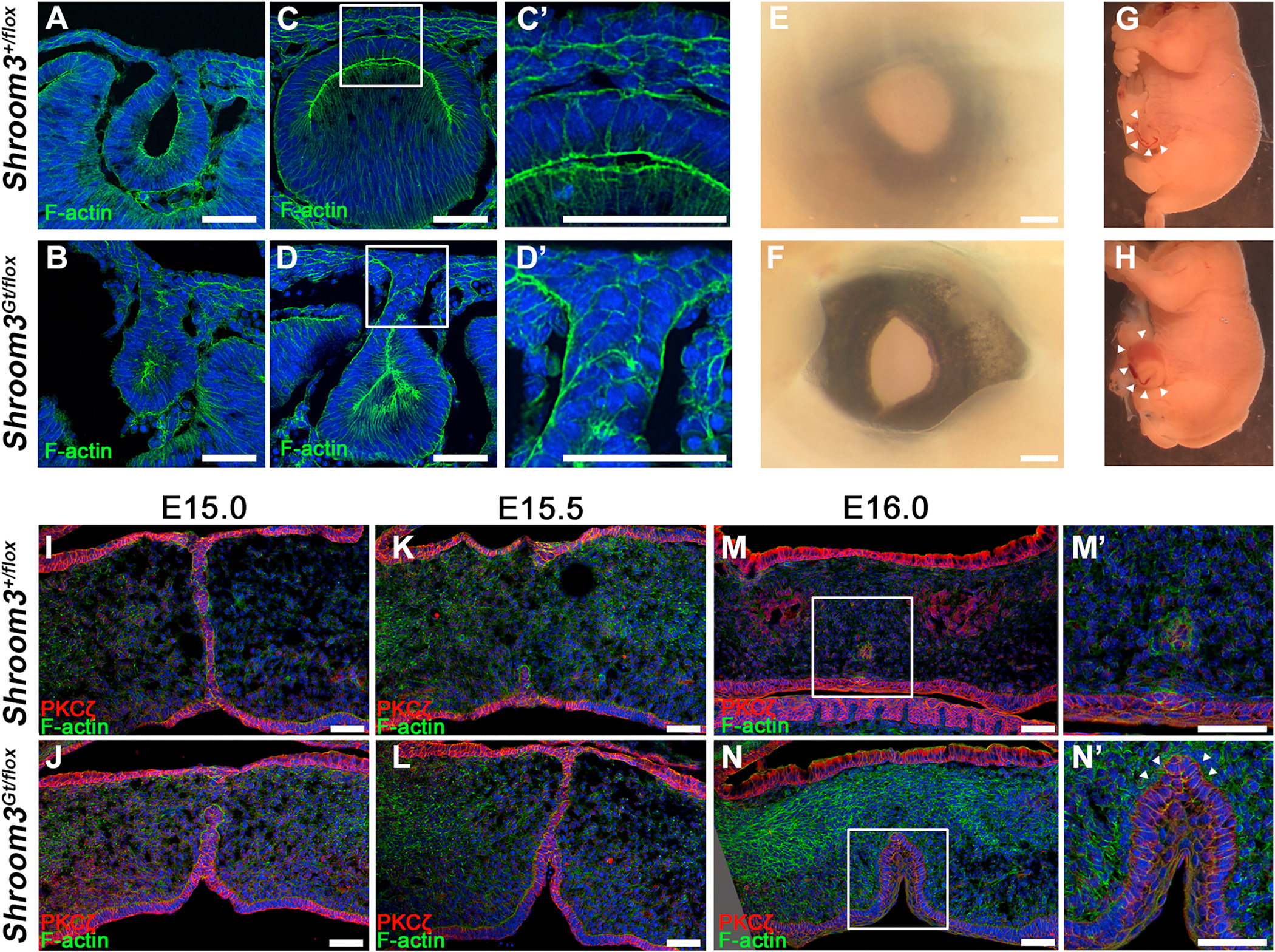
Shroom3 regulates fusion events in multiple tissues during embryonic morphogenesis. A-D) Coronal cryosections through the lens and presumptive cornea of control and Shroom3 mutant mouse embryos at E11.0 (A–B) and E12.0 (C–D) stained with phalloidin (green) and Hoechst (blue). The squared areas are magnified in the right panels. Note that although fusion can occur to produce a lens vesicle in control and mutant eyes, Shroom3 deficient lenses do not normally separate from each other and the epithelium appears disorganized. E-F) Whole E15.5 eyes from control and mutant embryos demonstrating that eye lid closure does not occur without Shroom3. This may be due to the inability of the eyelid margins to zipper up. G-H) Lateral view of the abdomen of control and Shroom3 mutant E15.5 embryos. Note that the ventral body wall does not close properly in the mutant embryos. Whereas control embryos display only a slight umbilical hernia at this stage (arrowheads in G), mutant embryos have herniation of the developing liver and intestines (arrowheads in H). I-N) Coronal cryosections through the secondary palate of control and Shroom3 mutant mouse embryos during the stages of tissue fusion at E15.0 (I–J), E15.5 (K–L), and E16.0 (M–N) stained with PKCζ (red), phalloidin (green), and Hoechst (blue). The squared areas in M-N are magnified in the right panels. Note that arrowheads mark a region of disorganized epithelium at the fusion point in the abnormal oral facing cleft in mutant embryos. The scale bars represent 50 μm in all panels. (For interpretation of the references to colour in this figure legend, the reader is referred to the Web version of this article.)

It is posited that the disruption of polarity occurs in Shroom3-deficient mutant embryos due to a failure of the epithelia to reestablish apical-basal polarity during the optic fissure fusion process (Fig. 8I). In order for fusion to occur, a subset of both presumptive neural retina (grey cells Fig. 8I) and RPE cells at the fusion point must break down their junctions with existing neighbors and reform them with new cells. During this process, cells at the fusion point will possess basal cell-cell contact with the cells of the opposing epithelium. Simultaneously, cells near the neural retina/RPE break point need to re-orient their apical membranes toward the newly created space (Fig. 8I, blue region). Because of the discontinuous nature of the apical markers specifically at the fusion point, we conclude that the reestablishment of apical-basal polarity during optic fissure fusion is Shroom3-dependent (Fig. 8I). Shroom3 is also expressed in many epithelial tissues where epithelial fusion occurs including the lens and neural plate (Hildebrand and Soriano, 1999; Plageman et al., 2010). To determine if the role of Shroom3 in epithelial fusion is universal we analyzed several places in the embryo where this takes place. As the lens pit forms a vesicle, the lens epithelium fuses and separates from the presumptive cornea at E11.0 (Fig. 9A and B). However, in the absence of Shroom3 the fusion process appears disorganized and the normal separation from the presumptive cornea at E12.0 fails to occur, and the lens remains attached to the surface ectoderm (Fig. 9C vs 9D). This region often contains cells that appear rounded without a clear apical-basal polarity (Fig. 9D). Fusion events are also required for eyelid formation and ventral body wall closure which are both disrupted by a lack of Shroom3 (Fig. 9E–H). The secondary palate also requires epithelial fusion, and in the absence of Shroom3 incomplete fusion occurs along with an inward bend that is similar to what is observed in the optic fissure (Fig. 9I–N). The orally facing epithelium appears to possess a region of cells without a clear apical-basal polarity at the apex of this fold (Fig. 9N, arrowheads). The failure of normal epithelial fusion in all of these tissues and the presence of disorganized cells at the fusion point in the absence of Shroom3 further supports its role in reestablishing apical-basal polarity during this process.

## 3. Discussion

The results described herein provide further mechanistic insight into two distinct steps of optic fissure closure that are Shroom3 dependent and necessary to prevent coloboma formation: the approach of the optic fissure margins and the reestablishment of apical-basal polarity. Our current understanding of the manner by which the optic fissure margins approach each other is somewhat limited but generally thought to be a consequence of tissue patterning and growth. But, cross-species analysis demonstrated that the margins of the optic fissure align and reorient themselves suggesting that a more active process may be involved (Bernstein et al., 2018). It is intuitive to consider that basal constriction, where cells reduce their basal surface area through actomyosin contraction, may be playing a role by reducing the surface area of the inner, lens-facing retinal epithelium and bringing the margins of the optic fissure closer together. While disrupting basal constriction does disrupt morphogenesis of the optic cup its role in optic margin approach is unclear and may only drive formation of the bilayer (Martinez-Morales et al., 2009; Nicolas-Perez et al., 2016; Sidhaye and Norden, 2017). Furthermore, we found that Shroom3 localization is limited to the apical junctions and is unlikely contributing to basal constriction. A role at the basal side of the cell should probably not be ruled out as Shroom3 can be recruited to the basal side of cells during basal constriction (Plageman et al., 2011b). Given that actin and myosin localization is relatively dynamic at the basal junctions of basally constricting cells (Nicolas-Perez et al., 2016; Sidhaye and Norden, 2017), it remains possible that the immunostaining was insufficient to detect transient or weak basal Shroom3 localization. Although potentially informative, the degree of basal constriction of NR cells was not tested due to the dense staining in histological sections of membrane and junctional markers as well as the F-actin containing axonal projections that begin to emanate from these cells at this age (Silver and Sapiro, 1981). Future experiments that genetically label individual cells with a fluorescent markers coupled with live imaging are promising avenues of further investigation.

The data presented here support a role for the geometry of cells within the outer curvature of the retina to drive the approach of the optic margins toward each other. When Shroom3 deficient, NR and RPE cells increase their apical dimensions causing the surface area of this epithelium to increase. Therefore, the failure of this tissue alignment prior to optic fissure fusion does not occur correctly and is likely a causative factor for the severe coloboma phenotype. Furthermore, the ability of the fissure margins to permit fusion in the absence of Shroom3 specifically in the neural retina implicates that Shroom3 function in the RPE may be sufficient for tissue alignment. By maintaining narrower cells by restricting their apical and basal widths, the RPE cells may be able to facilitate a smaller radius of curvature and align the margins at the appropriate point. It was unsurprising that the apical width of RPE cells are wider in the absence of Shroom3 but it is unclear why the basal side of RPE cells were likewise affected. It can be surmised that the alteration of the apical area may indirectly impinge on either the total volume of RPE cells and/or the junctional tension within basal junctions similar to what can occur in other epithelia (Barrera-Velázquez and Ríos-Barrera, 2021). It is also worth noting that the cell geometries measured here may not perfectly reflect what is occurring during morphogenesis. While the average shape is assumed from linear two-dimensional data, cells may vary in distinct ways that would only be appreciated from a three-dimensional analysis and/or a more regional characterization of cell shape changes and behaviors. Furthermore, these models rely on examples of cellular geometry of fixed tissues which may not always faithfully replicate what occurs *in vivo*. For example, the RPE cells often appear flatter and wider in some places and taller and wider in others. Changes in regional proliferation indices of cell populations not observed in this study such as toward the posterior optic cup could also indirectly affect morphogenesis. Indeed, RPE cell hyper proliferation can lead to the formation of a coloboma in mouse embryos (Sun et al., 2020). Additionally, epithelial curvature does not necessarily have to rely on mechanisms of apical/basal dimensions. The presence of scutoid shaped epithelial cells can theoretically induce epithelial bending without apical/basal constriction and are present in curved epithelia undergoing morphogenesis (Gomez-Galvez et al., 2018). However, what controls scutoid shape in epithelial cells is unstudied.

The alteration of RPE shape during optic cup morphogenesis is also not unprecedented. In zebrafish embryos, RPE cell flattening is thought to facilitate optic cup formation by potentially providing a propulsive force for the cell movements occurring within the optic cup as the bilayer forms (Cechmanek and McFarlane, 2017; Moreno-Marmol et al., 2021). Formation of the optic fissure margins depends on a flow of retinal neuroepithelial cells from the outer optic cup to the inner, lens facing layer in both zebrafish and chick embryos (Kwan et al., 2012; Heermann et al., 2015). Because perturbations of crucial signaling pathway components that regulate this epithelial flow can also lead to the formation of a coloboma, it may be inferred that this flow may not only form the margins but also induce their approach. Additional live imaging experiments that monitor epithelial flow during margin approach would be useful to support this inference. It is unclear from this study whether similar epithelial flow patterns are disrupted in Shroom3 mutants, but it would be interesting to determine whether the Bmp and/or hedgehog signaling pathways, which regulate this cellular movement, are also upstream of Shroom3-dependent shaping mechanisms.

Based on the results presented here, the failure of reestablishing apical-basal polarity at the fusion point in Shroom3 mutant embryos is thought to lead to a novel type of ventral optic cup abnormality, where an involution of neural retinal cells persists at the ventral side of the optic cup. To our knowledge this is the first time this phenotype has been documented during optic cup morphogenesis. However, it is postulated that it may be more common than historically reported due to a potential lack of histological analysis of somewhat normal-looking ventral optic cups without a disruption to the pigmented layer. Clinical cases of isolated retinal coloboma can occur independently of iris malformations (Lingam et al., 2021) and it remains possible that a failure of apical-basal polarity reestablishment during fusion may underlie a subset of these cases.

This phenotype also points to a novel function for Shroom3 in apical-basal polarity establishment. When tissue fusion at the optic margins occurs, the epithelial cells at the margins need to reshuffle into two distinct epithelial layers. During this time, a select number of cells at the margins known as pioneer cells, briefly attenuate apical-basal polarity and transiently alter gene expression to facilitate this process (Hardy et al., 2019; Eckert et al., 2020; Hardy and Rainger, 2023). The pioneer cells eventually reestablish apical-basal polarity and the data presented here suggest that this process may be Shroom3-dependent. The mislocalizaton of ezrin at this fusion point may point toward how Shroom3 directs polarity. Ezrin normally localizes apically to RPE cells and other epithelia, is required for normal optic cup morphogenesis in zebrafish, and functions to establish the apical domain of epithelial cells (Kivelä et al., 2000; Zhu et al., 2010; Sidhaye and Norden, 2017). Whether Shroom3 directly acts to sequester ezrin apically or does so indirectly is yet to be determined. A role for Shroom3 is also supported by a number of additional studies. The absence of Shroom3 in the intestinal epithelia disrupts the organization of villi and is required to maintain its pseudostratified nature, which is similar to what we observe at the fusion point of the optic margins (Grosse et al., 2011). Shroom3 can regulate microtubule organization in *Xenopus* neuroepithelial cells which may link microtubule-dependent polarity mechanisms with Shroom3 function (Lee et al., 2007; Meiring et al., 2020). Alternatively, the association of Shroom3 with F-actin and potentially Ena/Vasp (Hildebrand and Soriano, 1999; Hildebrand, 2005; Plageman et al., 2010) may also facilitate apical-basal polarity as the enrichment in actin has been implicated in driving polarization of epithelial cells (Baas et al., 2004; Galvagni et al., 2012). Identifying additional cellular mechanisms of Shroom3-dependent polarity regulation is an important avenue of future investigation.

Together, the findings presented here enhance our understanding of Shroom3’s role in optic fissure closure and its essential contribution to preventing coloboma formation. By elucidating Shroom3’s involvement in both the approach of optic fissure margins and the reestablishment of apical-basal polarity, this study opens new avenues for exploring the molecular mechanisms underlying these processes. Future research should focus on dissecting the complex cellular dynamics at play utilizing live imaging of epithelial flow patterns, cell shape regulation, and apical-basal polarity establishment during fusion. A more comprehensive understanding of Shroom3’s multifaceted role in ocular development is a promising way to further elucidate mechanisms of epithelial morphogenesis.

## 4. Methods

### Animal maintenance and use.

Mice were housed in in accordance with IACUC institutional policies. Mouse embryos were collected at distinct gestational ages by defining the day of detection of a vaginal plug as 0.5 [embryonic day (E) 0.5]. At specific gestational ages, embryos were removed by hysterectomy after the dams had been anesthetized with isoflurane. The following mouse lines utilized have been previously described: *Shroom3*^*Gt*^ (*Shroom3*^*Gt(ROSA)53Sor*^) (Hildebrand and Soriano, 1999), *mRx-cre* (*Tg(Rax-cre)1Zkoz*) (Klimova et al., 2013), Shroom3^R1838C^ (*Shroom3*^*m1Nisw*^) (Marean et al., 2011). The *Shroom3*^*flox*^ line was generated by Cyagen Biosciences Inc. In brief, a targeting vector was constructed such that exon 5 was flanked by loxP sitescausing exon 4 to be spliced into exon 6 upon recombination. Only the endogenous N-terminal 194 amino acids would be translated before prematurely truncated by the placement of a stop codon in frame which causes the resulting peptide to lack the functional C-terminal domains of Shroom3 (actin binding and rho-kinase domains). A neo cassette flanked by self-deletion anchor (SDA) sites and a *Diphtheria* toxin A (DTA) sequence were included in the targeting vector for positive and negative selection, respectively. The vector sequence was confirmed by restriction digest and Sanger sequencing. The vector was linearized and purified prior to electroporation into C57BL/6 embryonic stem (ES) cells and subjected to G418 selection (200 μg/mL) 24 h post electroporation. 182 clones were screened via PCR for homologous recombination and 6 were expanded and characterized by Southern blot analysis. ES cells were injected into blastocysts and implanted into surrogate females. F0 mice were interbred to produce F1 pups and SDA sites obviated the need for removal of the neo selection cassette following founder establishment. Animals were genotyped using tail DNA with the following primers and PCR conditions: 5′-CCAGGAAGGTTGCCAGAGTCTAGCT, 5′-CTGTCCGTTGTGGATGCTCGTG; 94 for 3 min (94 for 30 s, 60 for 30sec, 72 for 30sec)x33cycles 72 for 30sec which generates a 160bp fragment for the wild-type allele and a 194bp fragment for the floxed allele. Successful recombination of the floxed allele was determined from crossing to the mRx-cre line and isolating genomic DNA from the retinas of compound heterozygotes and performing PCR using the following primers and conditions: 5′-CCAGGAAGGTTGCCAGAGTCTAGCT +5′-gctggccttga acgcacagag 94 for 3 min (94 for 30 s, 60 for 30sec, 72 for 30sec) yielding 377 bp band when recombination is present. The reporter line utilized for tracking cre activity was B6.129(Cg)-Gt(ROSA)26Sortm4 (ACTB-tdTomato,-EGFP)Luo/J and acquired from Jackson laboratory (Strain #007676) (Muzumdar et al., 2007).

### Histological assays.

Embryonic tissue was prepared for histology following two rounds of sucrose infiltration (15 % and 30 % sucrose in 1xPBS) allowing the embryonic tissue to sink between steps, embedded in OCT media, and cryosectioned with a 10 μm thickness. For X-gal staining, embryos were fixed (1 % formaldehyde/0.2 % glutaraldehyde, 2 mM MgCl_2_*6H20, 5 mM EGTA and 0.01 % NP-40 in 1xPBS) for 45 min at 4 °C under gentle rocking, rinsed with 0.02 % NP-40/1xPBS twice for 15 min, and incubated in X-gal solution (5 mM K_3_Fe(CN)_6_, 5 mM K_4_Fe (CN)_6_, 1 mM MgCl_2_*6H20, 0.01 %NP-40, 1 mg/ml X-gal, 1xPBS) for 14 h at 37 °C with gentle rocking. The stained embryos were cryosectioned and imaged. For immunoflourescent labeling, embryonic tissues were fixed in 4 % paraformaldehyde in 1xPBS, embedded as above, and prepared using protocols as previously described (Houssin et al., 2020). The specific primary antibodies utilized and their dilution utilized were as follows: Ezrin/1:200 (3145S, Cell Signaling), Laminin/1:250 (SAB4200719, Sigma Aldrich), Mitf/1:250 (Sigma Aldrich HPA003259), Otx2/1:250 (Novus Biologicals/AF1979), phospho-histone H3/1:1000 (EMD Millipore/07–424), PKCζ/1:1000 (Santa Cruz Bio/sc-216), ZO-1/1:500 (Invitrogen/33–9100). A custom rabbit polyclonal Shroom3 antibody was generated by inoculating rabbits with a recombinant peptide spanning amino acids 1562–1968 of the mouse Shroom3 protein sequence (GenScript USA) and utilized at a 1:500 dilution. Fluorescently labeled Phallodin/1:200 (Invitrogen/A12379) and Hoechst 33342/1:1000 (Sigma, B-2261) was utilized to detect F-actin and nuclei, respectively. A combination of Alexa Fluor secondary antibodies conjugated with 488, 568, 594, or 647 fluorophores (Invitrogen) were utilized to detect the primary antibodies. Fluoro-gel medium (Electron Microscopy Sciences) was used to mount coverslipped sections and the sections were imaged with a Zeiss Axio observer inverted microscope equipped with a fluorescent light source and a 40x Plan-Apochromat objective or a BC43 spinning disc confocal microscope equipped with four laser lines and 20x Plan Apo LD air/60x Plan Apol LD oil immersion objective.

### Western blot:

Protein lysates were isolated from E15.5 mouse embryo kidneys/intestines in RIPA buffer and homogenized. Following centrifugation, the supernatant was boiled in 2x Laemmli sample buffer and beta-mercaptoethanol at 90 °C for 5 min. The lysates were run in a 10 % acrylamide Mini-Protean TGX Precast Gel (Bio-Rad) and transferred onto a PVDF membrane using a semi-dry transfer. The membrane was immunolabeled using a custom Shroom3 antibody (described above) at a 1:500 dilution at 4 °C overnight and a mixture of rabbit-HRP and StrepTactin-HRP at a 1:2500 dilution (BioRad) at room temperature for 1 h. The ECL reagent kit (5 min at room temperature) and a Bio-Rad Chemidoc imager were used to detect the protein.

### Quantitative analysis:

The apical area of individual NR cells was determined using images of tangential sections or whole mounted tissue stained with phalloidin and the area tool on ImageJ. Patches of 50–60 cells near the nasal and temporal walls of the optic cup were measured from images of 4–6 different E12.5 eyes. To determine the thickness of the pseudostratified NR cells, sagittal sections were analyzed and the distance between the apical and basal side of the cells were measured at 5 evenly distributed points along the dorsal and lateral sides utilizing the line tool of ImageJ, avoiding the ventral side where the variability is at a maximum and ensuring the line is parallel with the apical-basal axis. RPE cells were similarly measured but 15 evenly distributed points were analyzed instead. 4–7 different E12.5 eyes from distinct embryos were analyzed for the RPE/NR thickness analysis. The distance along the outer apical curvature of the NR (reported as the apical length) was determined from sagittal sections of 7 different eyes from different embryos chosen based on the presence of a mid equatorial lens cross section and utilizing the freehand line tool to calculate the distance. To quantify the mitotic index of NR cells, images of sagittal sections of E12.5 eyes were compiled from multiple 40x widefield images that were stitched together utilizing the automerge function on Adobe Photoshop. Positive PH3 cells, were counted using the Cell Counter function on ImageJ. Positive cells were counted if located within the neuroepithelium and co-localized with nuclei. Damaged (e.g. missing) and folded tissue were also excluded from total area outlines and calculations. 7 different eyes from different embryos were analyzed and the number of cells per unit area was calculated. To determine the average shape of RPE, the total number of cells were manually counted along the entire sagittal section and the apical and basal distance of the RPE cell layer was measured using the freehand line tool in ImageJ. 3 sections each from 4 (*Shroom3*^*Gt/flox*^ analysis) or 5 (*mRx-cre; Shroom3*^*flox/flox*^ analysis) distinct embryos were analyzed, excluding damaged regions and the ratio of apical or basal distance per cell was determined. The means and distribution of the data are presented in the text and plots within the figures. Significant differences were determined utilizing student t-tests.

## Data Availability

Data will be made available on request.
